# Visualization of text on bowed sheets via High-resolution 3D-Magnetic Resonance Micro-imaging for potential reading of closed books: the proof-of-concept

**DOI:** 10.1038/s44172-026-00614-7

**Published:** 2026-02-25

**Authors:** Andreas Georg Berg, Alexander Karl Seewald

**Affiliations:** 1 Center for Medical Physics and Biomedical Engineering, Medical University of Vienna, Vienna, Austria; 2 High Field Magnetic Resonance Center, Medical University of Vienna, Vienna, Austria; 3 Seewald Solutions GmbH, Weißkirchen an der Traun, Austria

**Keywords:** Magnetic resonance imaging, History, Microscopy, History

## Abstract

Non-invasive Magnetic Resonance Imaging on clinical MR-scanners at spatial resolution ≂1mm^3^ could in principle be used also for insights in the structure of valuable ancient objects like books if the spatial resolution could be improved. We demonstrate, that Magnetic-Resonance-Microscopy is even able to visualize printed letters at thickness < 30 µm on superposed paper-sheets. The physical-technical methodology is based on prototype hardware installed as insert on a human MR-scanner relying on strong magnetic and gradient-fields with sensitive radiofrequency-sensors. A negative contrast mechanism, adding MR-visible chemically-inert liquid, is necessary, its removal being potentially harmful to valuable paper sheets. For visualization of text firstly the letter thickness (≂20 µm) and exact positions were determined before adjusting optimized resolution and field-of-view (FOV) in measurement and reprocessing of 3D-data for slice-positioning in the plane of the paper sheets. The advantages of a semi-automatic data processing method for visualization on the bowed paper-sheets are demonstrated. In contrast to micro-Computed tomography based on absorption contrast, MR-microscopy can visualize non-metallic printed letters and offers higher spatial resolution than Terahertz imaging. As a preclinical MR-imaging tool (with limited FOV) it is more widely available than neutron-tomography. The reported MRM-technology might be also of interest for radiological high-resolution applications such as MR-based histology.

## Abstract (web summary)

Can techniques for non-invasive diagnostic imaging on humans be used for reading of letters in closed books? Andreas Georg Berg and Alexander Karl Seewald demonstrate that Magnetic-Resonance-Microscopy established on a High-field human MR-scanner can visualize printed letters on superposed paper sheets.

## Introduction

Non-invasive imaging methods based on, e.g., X-ray, Ultrasound and Magnetic Resonance are not only necessary for the diagnostic imaging on humans but represent valuable visualization tools in non-destructive material analysis. They have also been applied for the analysis of mummies and archeological objects, e.g., the famous finding of an arrowhead, which killed “Oetzi”, a mummy frozen in the alps more than 5000 years ago^1^. But the applications are limited by resolution and contrast available on clinical scanners to a voxel-size of about 1 mm^3^. The imaging of letters, for instance in ancient text books, appeared to be inconceivable on human MR-scanners especially due to the non-water containing paper sheet material used and the related very short decay times of MR-signals. In addition, printed letters in books feature a typical thickness of the pigment layer between 0.5 µm and about 14 µm for printable inks^2^. Therefore, the imaging of small-sized print letters, for instance, in ancient text books, appeared to be not possible on human MR or CT- tomographic scanners, especially due to the missing spatial resolution. Actually, µ-CT scanners with limited field of view (several cm) became recently available. Text components on an ancient scroll with ink based on a Gall apple iron mixture could already be visualized^3^. However, the visibility of letters by µ-CT has been relying mainly on the absorption contrast of the used X-rays, for which the presence of elements with high atomic charge number Z as present in iron is crucial. Many inks are based on non-metallic pigments using resin or rubber materials as binder material, which contains hardly any element with high Z.

Magnetic resonance imaging (MRI) is known to offer also contrast for soft tissue (i.e., no bone). containing only small concentrations of high Z elements. There are two main restrictions in MRI, which appear to not allow for the visualization of the tiny, ink-covered spots on sheets:

1. the spatial resolution even on Ultra-high field MR-scanners for human applications has been restricted to voxels of about (400 µm)^3^, much larger than the thickness of printed letter structures^4^;
2. the pigments and resin/rubber binders in the dry ink cannot be directly detected as the signal relaxation (transverse relaxation time *T*_2_) after excitation is too short (µs) for getting any signal from the involved molecules.

We wish to demonstrate within this manuscript that the visualization of small-sized letters with thickness of the printed ink of about 28 µm is in principle possible using additional hardware equipment on a human scanner, an added, chemically inert, MR-active liquid and MR pulse sequences and 3D MR-protocols optimized for highest spatial resolution.

We will firstly describe the principal conceptual ideas for removing the apparent two limitations in standard high-resolution MR-imaging. We then will show our first methodological approaches on single large-scale letters at different spatial resolutions and finally will present the results of 3-dimensional MR-microscopic data sets on full text passages on stacked paper sheets, similar to the way paper sheets are arranged in books. Even short word patterns printed on a modern color printer could be visualized using the reprocessing of the 3D MR-data set for a planar layer reconstructed to the surface of the printed paper sheets.

Full sentences with improved contrast were reconstructed using parametric algorithms for the visualization of even bowed paper sheets as documented in the related section “Improved, flattened visualization of the printed text on bowed paper sheets”. This approach avoids extensive manual adjustment of reading slices approaching a final goal of automatic deciphering of closed books and also improves reconstruction quality of text patterns on bowed paper sheets.

## Materials and methods

### General aspects and physical-technical concepts for solving technical challenges

**MR-visibility of the printed material:** Solid, dry materials cannot be detected directly by MRI as the molecules of the solid components carrying protons used for high-resolution MRI decay very quickly with *T*_2_ in the order of µs, which is too short for imaging encoding. Therefore, an indirect negative contrast has to be established by adding an MRI active liquid or gas with high signal intensity, which features no or low impact on the paper and printed letter. **Spatial resolution:** the spatial resolution on MR-scanners for imaging of humans with large Field-Of-Views (FOV) – typical for books (e.g., about 40 × 40 × 40 cm^3^) – is limited to a voxel-size (VS) of about (100 µm)^3^ even on Ultra-High-Field (UHF) MR-scanners (B ≥ 7 T)^5^. This essential limitation is overcome (partly) within our presented approach as a proof of concept using an additional prototype microimaging insert to a human UHF MR-scanner. The technical solutions rely on the high magnetic field (B > 3 T), high sensitivity of the MR radiofrequency (RF) detectors, time efficient spatial encoding, averaging and high magnetic field gradient strength G. **2D reading on bowed paper sheets:** As the exact position of the letters is not known a-priori before visualization, first 3-Dimensional (3D)-data sets at high spatial resolution have to be obtained, from which the characters and letters have to be identified with contrast against non-information carrying parts of the 3D-data sets. This problem is approached by using the imaging shape of the paper sheet as a guide for accurate positioning/tilting a planar layer for optimum contrast reading. We demonstrate here:

- the result of a simple approach by manual positioning of plain slices in the 3D-MRM data set;
- the result of an advanced semi-automatic approach with 2D visualization along curved virtual planes along the bowed paper sheets (see section *“Improved, flattened visualization of the printed text on bowed paper sheets*”).

### Sample preparation

Single printed Laser toner letters (black) are reported for a thickness (th) of the printed pigment/polymer material of th ≅ 2.5 µm^6^. The lateral letter size varies in the mm range and therefore appears to be available for MR-microscopic methods^7,8^, but the MR-visualization of the thin pigment print layer against the paper sheet seemed to be impossible even with the smallest voxel sizes (VS ≅ (3 µm)^3^) available on MR hardware optimized for highest spatial resolution in MRM^9^ with very small FOVs. Printing of all colors (C, M, Y, K) at same position on one sheet of paper resulted in an increased thickness of printed layer th_CMYK_ = 7.5 µm, which appeared still not available to the MR-microscopy prototype at the UHF human MR scanner^8,10^. We, therefore, prepared different sets of multi-fold over-printing at the same position ranging for two-fold (2x) to eight-fold (8x) CMYK layer printing at same position. The procedure should result in nominal printing thickness from 15 µm (as indicated for the upper range of printing thickness for standard modern printers^2^) to 60 µm for demonstration of the principal potential for MR-visualization (see Table 1).

**Table 1:** Sample sets printed for MRM visualization with different letter size and thickness by multifold overprinting (multiplicity)

Sample set	lateral letter size	letter-printing multiplicity	MR-active liquid
Set 1 single letters (“BC2”)	2.3 × 2.8 mm^2^	8 x	none (UTE imaging)
Set 2 single letters (“AB”)	2.3 × 2.8 mm^2^	8 x	tap water with contrast agent (CuSO_4_ · 5H_2_O; 8 mMol/L)
Set 2 single letters (“BC”)	2.3 × 2.8 mm^2^	8 x	silicone oil
Set 3 text patterns in 9 superposed paper sheets	1 × 1.3 mm^2^	2 x, 4 x, 8 x (15, 30, 60 µm)	silicone oil

For checking the potential for direct observation with Ultrashort Time Encoding (UTE imaging)^11,12^ sample set 1 was arranged without MR-active liquid. Sample set 2 was prepared by adding tap water in a small glass tube using a contrast agent as add-on (copper sulfate, CuSO_4_· 5 H_2_0; 8 mMol/L) for reducing *T*_*1*_ in the interest of short measurement time (repetition time TR « *T*_1_). Due to best experience with initial experiments most of the sets were prepared with silicone oil (Polydimethylsiloxane, Elbesil B100 Silikon Profis Quax GmbH Obernburg/Germany) as an MR-active liquid (Table 1). The silicone oil was added after positioning of the stacked paper sheets in the syringe. It entered easily the tiny slits between the paper pages with hardly any left oxygen bubbles as observed for water. The silicone oil featured low viscosity (*η* = 100 cst) and is known for being rather chemically inert^13,14^.

After checking the potential for the visualization of single letters, whole virtual sentences (text pattern) composed of maximum nr. of different letters and numerals were printed in different multiplicity on several sheets of paper (Table 2).

**Table 2:** Sample Set 3: text patterns for the 3 subsets of paper sheets with different printing multiplicity used for 3D MRM

Text pattern	Subset 1 sheet 1-3	Subset 2 sheet 4-6	Subset 3 sheet 7-9
THE QUICK BROWN FOX JUMPS OVE R THE LAZY DOG 01234	2 x	4 x	8 x
FIX SCHWYZ QUÄKT JÜR GEN BLÖD VOM PAß 0123456789	2 x	4 x	8 x
FRANZJAGT IMKOMPLETT VERWAHRL OSTENTAXI QUERDURC	2 x	4 x	8 x

**Fig. 1:**
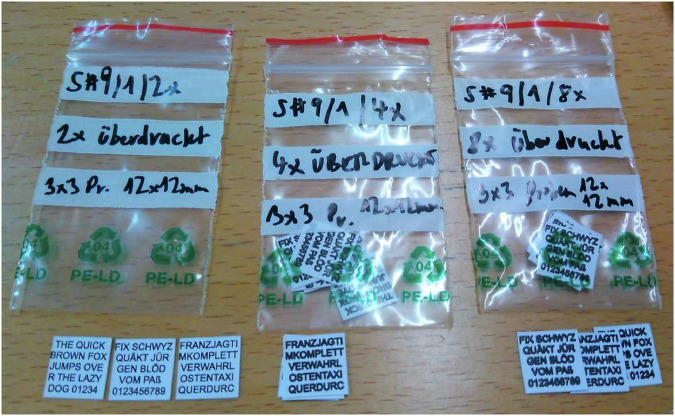
Photo of sample set 3 consisting of 3 subsets with different factors for multifold printing (multiplicity). left: 2x printing in color mode (CMYK); th ≃ 15 µm; center: 4x; th ≃ 30 µm; right: 8x, printing thickness (th ≃ 60 µm); the increased thickness results in appearing bolder.

A photo of the samples used with different printing multiplicity is shown in Fig. 1.

### MRM-microscopy apparatus, MR-pulse sequences and -protocols

#### Challenges and concepts

The physical-technical demands refer mainly to the small voxel volumes necessary for identification of the very thin letter structure, which represent challenges for the sensitivity of the RF-detectors and the spatial encoding in the direction of the letter thickness^7^. These challenges have been addressed using:

- prototype hardware add-ons on an Ultra-High Field (UHF) human MR-scanner (*B* = 7 T); These are mainly relying on a significantly stronger magnetic gradient field than available on a clinical MR-scanner (factor 10) and a sensitive RF-detector with small size adapted to the sample diameter.
- a time efficient 3D-FT pulse sequence and MR-protocols adapted to the MR-active liquid along with averaging of signal for Signal-to-Noise-Ratio (SNR) improvement.

Details of the sensitive hardware set-up are reported in the attached Supplementary Materials and Methods file in section *MRM-microscopy apparatus, MR-pulse sequences and -protocols*.

## Results

### Direct letter visualization and tap water with contrast agent approach for visualization

The direct visualization of the solid pigment letter elevation appeared unlikely due to the expected short *T*_2_-relaxation time of the corresponding MR-signal, but we wished to check for the actually printed CMYK inks using special MR pulse sequences optimized for Ultra short Time Encoding (UTE). However, this approach failed due to the low RF-signal, a consequence of the fast signal decay and small material volume.

The next approach, using tap water with an add-on contrast agent (CuSO_4_ · 5H_2_O) for reducing the measurement time, was more successful: The paper sheet surfaces could be well resolved at small voxel volume (VS = 59 × 59 × 60 µm^3^). However, the MRM image was severely distorted by a multifold variety of microscopic air bubbles attached to the surface of the paper sheet. No letter structure could be identified.

### Sample Set 2 using silicone oil as MR-active medium: Measurement of letter thickness and visualization of single letters

We changed the MR-active liquid to silicone oil for avoiding air bubbles. At first the available contrast and the spatial resolution with different MR-pulse sequences and parameter weighing (e.g., *T*_2_, *T*_2_* and *T*_*1*_) with gradient and spin echo sequences had to be checked. Gradient echo sequences appeared to be sensitive to the tiny microscopic air bubbles still left on top of some paper layers, which led to susceptibility related artifacts. Therefore, time efficient Turbo-spin echo sequences were chosen.

**Fig. 2:**
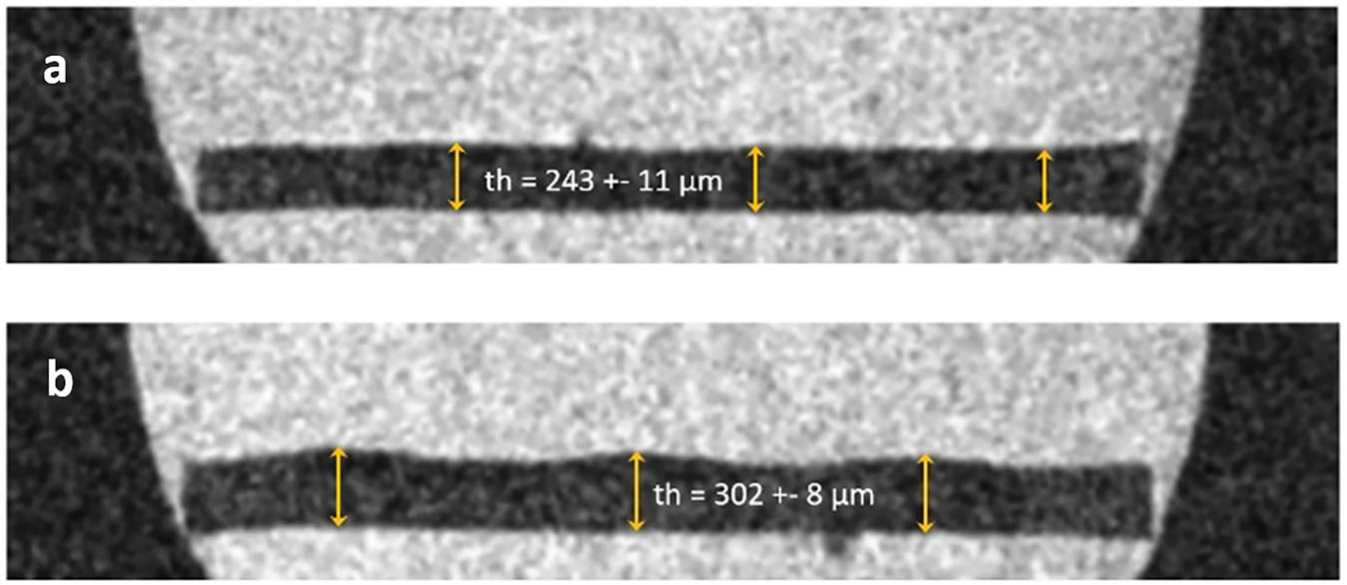
Sagittal MR images (silicone oil added) sectioning the single paper sheet (MR-protocol: 2D-Localizer_sag_; VS: 23.44 × 23.44 × 200 µm^3^). **a** (top): slice positioned in an area of missing printer ink. The thickness of the paper sheet is determined to be th_p_≅243 ± 11 µm. **b** bottom: slice positioned in an area of 3 elevations generated by the printed letters along a line crossing the B and the C letter. Note the negative contrast, i.e., the printed ink itself gives no MR-signal similar to the paper sheet. The height of the ink is not homogenously distributed. The maximum thickness of the ink in this 8-fold printing can be determined from the MR-microscopic images to be th_ink_max_ = 59 ± 14 µm as difference between the maximum averaged elevation th=302 µm in the region of the printed letters (yellow arrows) to the paper thickness outside of the letters (th ≃ 243 µm).

As a first test large letters (B,C, height h_L_ ≅ 2.4 mm) with 8 layers of toner material (CMYK, thickness th ≅ 56 µm) have been MR-scanned using an image layer rectangular to the paper sheets (Fig. 2).

These short (TM < 2 min) test images (Fig. 2) already indicated that the MR-active mobile liquid molecules did neither penetrate into the print material area, nor to the paper sheet; image contrast between the printed letters may be obtained and the maximum slice thickness for reading in-plane MR-images should be smaller or at least close to the max. letter thickness of 58 µm.

**Fig. 3:**
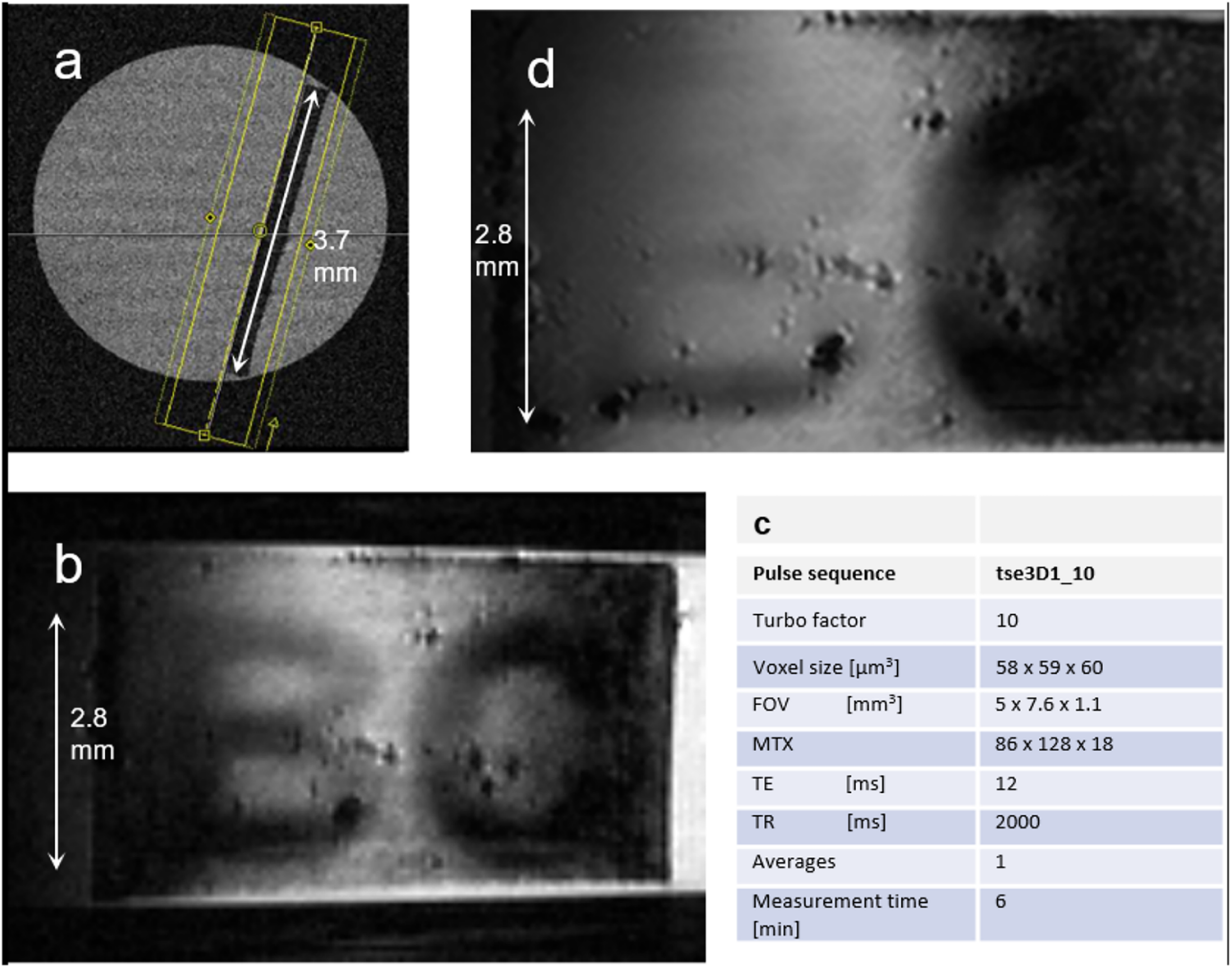
MR-microimaging of single letters. **a** Sagittal MR image showing the inclination of the paper sheet against initial main y-axis of the gradient coordinate system of the MR-scanner and the result of the adjustment procedure for MRI with slices in the plane of the printed letters. **b** MR image positioned in the plane of the paper sheet. (VS:58 × 59 × 60 µm^3^): The letters B,C (8x printing) can be read, although inhomogeneities in contrast (left-right vs. center) due to non-perfect slice positioning are visible. The tiny dark spots are magnetic susceptibility artifacts in MRI originating from small microscopic air bubbles attached to the surface of the paper sheet. **c** MR-measurement parameters for Fig. 3b. **d** MRM image with VS = 38 × 40 × 42 µm^3^. Though the 3D resolution is nominally sufficient and even better than the post-aligned data sets (**b**) the reading of neighboring letters is difficult due to non-perfect pre-alignment of the measurement slices to the paper layer.

Next, 3D imaging on single letters with slices in the printing plane of the letters was performed: a sagittal slice (Fig. 3a) along with localizer orthogonal transverse images were used for careful positioning in-plane (coronal type) slices (Fig. 3b) of a 3D-TSE pulse sequence with thin slices (th = 60 µm) by rotation around two orthogonal axes to the plane of the paper sheet.

The imaging result of the corresponding 3D TSE sequence is shown in Fig. 3b. These first imaging results already indicate the potential of MRM for reading printed letters, though the printed letter thickness of about 58 µm outranges that of standard printed letters evidently. The positioning of measurement slices in the MR protocols to the plane of the paper using localizer MR sequences might be not sufficient. An example is shown in Fig. 3d. Though the MR-protocol has been improved with reference to Fig. 3b from VS ≅ (60 µm)^3^ to a VS ≅ (40 µm)^3^ the obtained optimum image allows only for clear deciphering of one letter (C). The neighboring letter “B” is already appearing hyperintense and consequently might not be deciphered due to the visualizing layer being positioned only partly over the elevated printer material with thickness ≅50 µm.

**Fig. 4:**
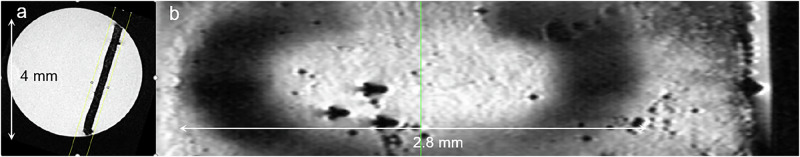
MRM-images of the isotropic Ultra-high-resolution-protocol. **a** (left) Sagittal MR view directly visible from the multi-slice 3D-iso20 protocol (see Supplementary Table S1). **b** right: Coronal type MR image calculated from the 3D-data set after positioning the displayed slice in the plane of the paper sheet (MR-protocol: 3D-iso20; VS = 19.5 × 19.5 × 20 µm^3^, MTX = 256x256x192). The letter C can be read with improved clarity.

Can the 3D MRM protocol be improved, such that arbitrary slice positioning with an isotropic voxel size significantly less than 58 µm can be achieved? We dealt with this question by changing the MRM protocols for highest resolution with expected limitation in signal-to-noise ratio (SNR) choosing the main slice orientation orthogonal to the paper sheet plane. Figure 4 left shows an image, sectioning the paper sheet with single letters in an area of the printed letter “C” as a result of the high-resolution 3D-TSEhres MRM protocol (3D-iso20) with an isotropic voxel size of about (20 µm)^3^.

### Sample Set 3: imaging of short sentences printed on stacked paper sheets

**Fig. 5:**
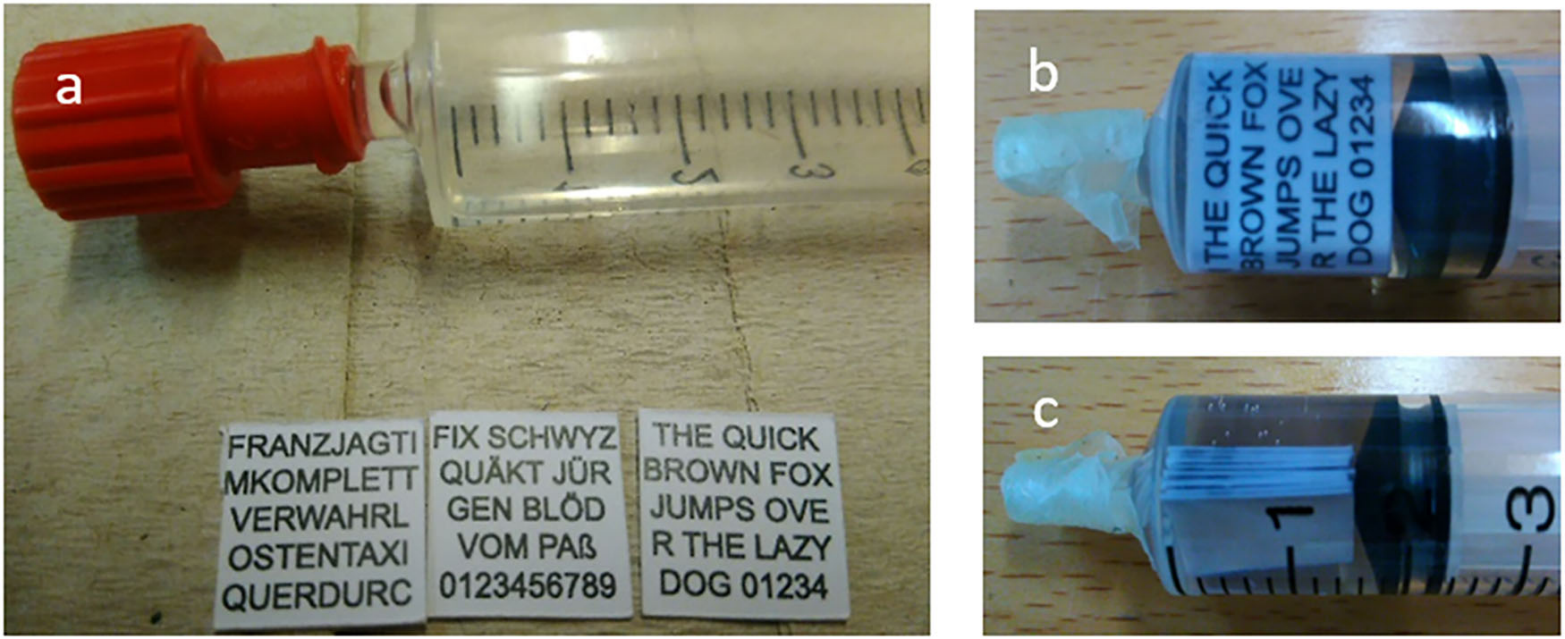
Photos of preparation of the 3 subsets of stacked text patterns. **a** Syringe container with chemically inert MR-active liquid and 3 paper sheets with different text patterns (2x printed layers of the CMYK toner material). Volume numbers are indicated in ml. **b** Frontal view of the 9 printed sheets in the liquid container, **c** Lateral view indicating the position of the 9 stacked paper sheets on top of each other. The unit 1 indicates 1 ml of volume in the container.

A stack of 9 paper sheets (see photo in Fig. 5c) has been prepared consisting of 3 subsets with different multiplicity of overprinting at the same position: subset 1: 2x printing; subset 2: 4x printing; subset 3: 8x printing. Each subset contains paper sheets with different text patterns comprising most of the different letters of the alphabet and numbers. Table 2 summarizes the different features of the 9 paper sheets arranged to the subsets.

**Fig. 6:**
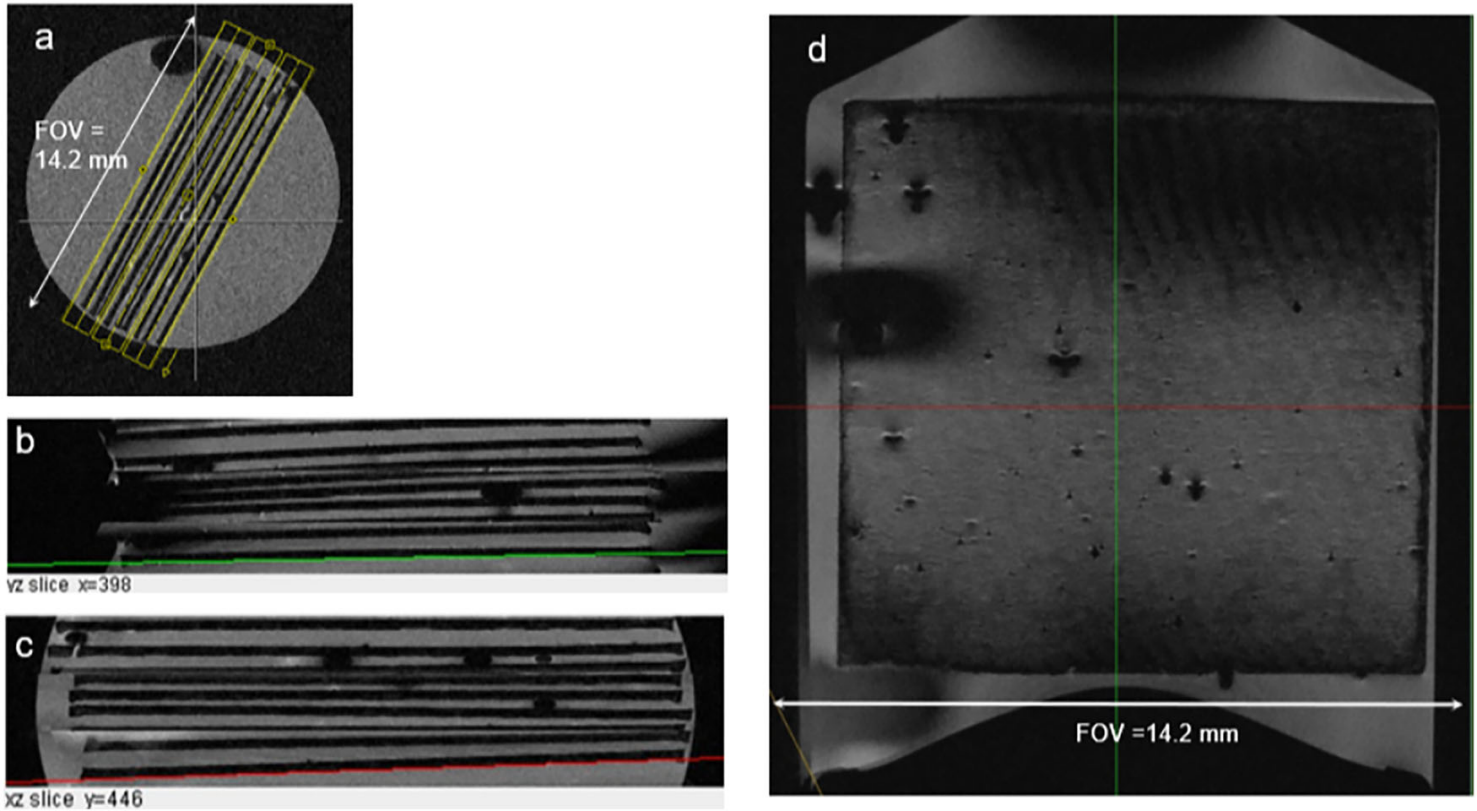
Multi-slice view of the high-resolution 3D- MRM data set (MRM-protocol: 3D-iso20_large_) for subset 1, sheet no. 1, 2x print. **a** MRI localizer image indicating the position of the high resolution 3D data set of Fig. 6d and Fig. 7 with three slabs with slice selective RF- excitation. **b** Sagittal view (yz-slice). The view is used for adjusting position and inclination (green line at bottom) of the final slice for visualization of the text pattern on top of paper sheet 1 (2x print, th ≃ 14 µm, Fig. 6d). **c** Transverse view (xz) for adjusting slice position and angle (red line at bottom) for best visualization of text pattern. **d** MRM slice view adjusted with viewing plane in the surface of paper sheet 1. (“THE BROWN FOX JUMPS OVER THE LAZY DOG 01234”). The text pattern can only partly be recognized. Note the problem of adjustment of the plane viewing slice to the curved hyperplane of the paper sheet, indicated by the darkened areas at bottom and top with imaging slice in the area of the dark solid paper.

**Fig. 7:**
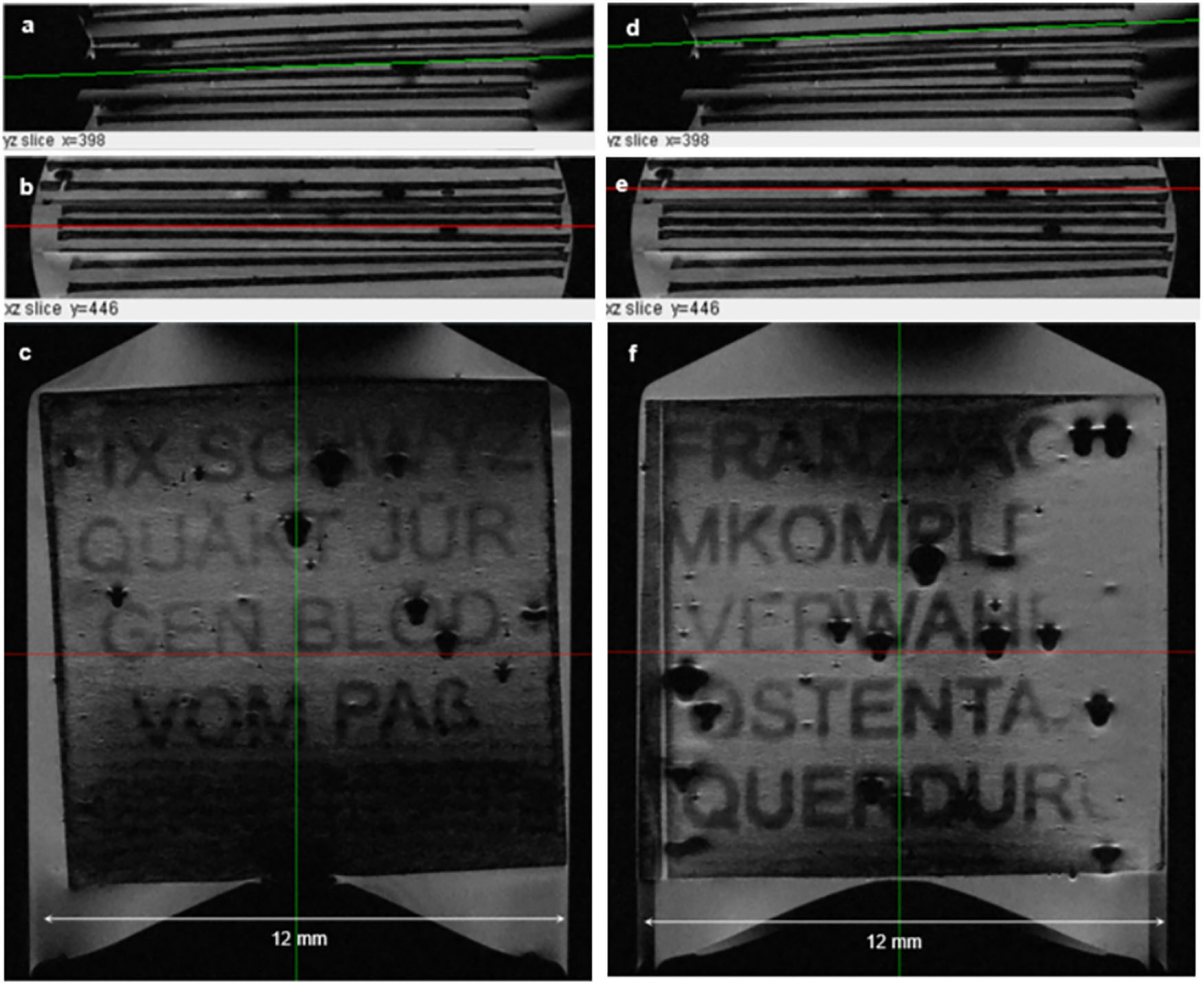
Localizer and slice view of the high-resolution 3D- MRM data set in the area of paper sheets with 4x and 8x printing. **a** Sagittal view (yz-plane) with green line indicating slice position of **c**. **b** Transverse view (xz) for adjusting slice and angle of the plane viewed in 6c (red line). **c** MRM slice view adjusted with viewing plane in the surface of paper sheet 5 (4x printing). The text pattern (“FIX SCHWYZ QUÄKT JÜRGEN BLÖD VOM PAß”) can be recognized much better than for the 2x printed letters (Fig. 6d). **d** Sagittal view (yz-plane) with slice position of **f** (green line). **e** Transverse view (xz). The plane viewed in 7 f.is indicated by the red line. **f** MRM slice view adjusted with viewing plane in the surface of paper sheets with 8x printing. The text pattern (“*FRANZJAGTKOMPLETTVERWAHRLOSTENTAXIQUERDURC*”) can be recognized best. Note the dark black spots due to microscopic air bubbles giving no signal and inducing some slight MRM distortions with bright rims.

**Fig. 8:**
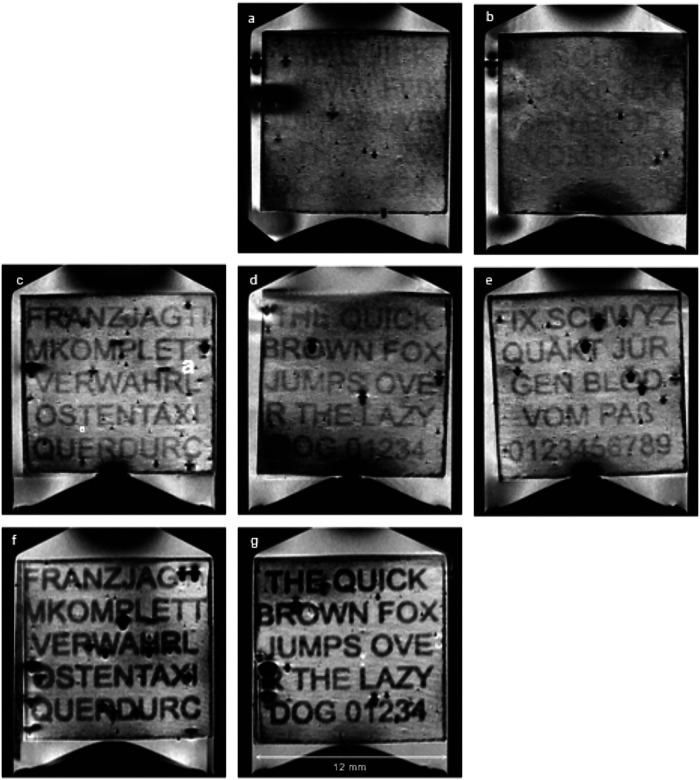
MRI-scanned book pages from a nine-page sample. Page reconstruction by cubic model (Eq. (3)). Toner layer thickness for the different number of layers: **a**, **b** first row *th*_2_ = 14 *µm* (two layers), **c–e** second row *th*_4_ = 28 *µm* (four layers), **f**, **g** third row *th*_8_ = 56 *µm* (eight layers). Each column corresponds to one distinct test sentence. The missing pages were omitted in the selection of MR-slabs and therefore could not be processed. Sheet numbers from top left to bottom right according to positioning (Fig. 9): 3 (missing),1,2; 6,4,5; 9,7,8 (missing). All images were automatically adjusted for white balance, which slightly improves contrast compared to Fig. 7c, f.

A 3D MRM protocol adapted to the volume of the 9 paper sheets has been set-up on the Microscopy insert for offering a high isotropic resolution with interpolated voxel size of about (20 µm)^3^ (see Figs. 6–8). MRM protocol parameters are listed in Supplementary Table S1. For time efficient 3D-imaging with small slice thickness the Turbo-spin echo sequence has been set-up combining slice selective encoding for 3 slabs with 64 phase encoding steps within the slab. Figure 6a indicates the positions of the slabs.

This combination of two different spatial encodings results in some small spatial distance between the slice-selected slabs and the two slight discontinuities seen in the orthogonal cross sections of Fig. 6b and 6c. In order to achieve a high SNR, the measurement was performed with high averaging (nr_av_ = 9) within an automated measurement lasting about 37 h. The 3D-image data set in DICOM format obtained from the standard *Syngo* Siemens user interface is arranged in 192 slices and was directly read in by a 3D processing open software tool (ImageJ)^15^. Using the standard plug-in, *3D-Volume viewer*^16^ displaying orthogonal views of the 3D data set is possible and allowed for the adjustment (Fig. 6b–d) of the position and inclination of the 2D-image slices for best viewing of the printed text on the slightly bowed paper sheet with 2-fold printing (Fig. 6d). The text pattern can only partly be recognized (e.g., “brown fox”) due to misalignment and relatively large slice depth (20 µm) with comparison to the letter thickness of estimated 14 µm. The MRM images for the other 2x printed text patterns appear similarly difficult.

Figure 7 shows the results of text patterns with 4x and 8x printing. The visualization of text is distinctly improved as a consequence of the increased letter thickness (≅ 28 µm for 4x printed text pattern in Fig. 7c) with reference to the voxel size (VS ≅18 µm).

However, the bottom part appears hypointense due to the slightly bowed paper surface. Finally, slice images of the third text pattern printed with multiplicity 8x have been calculated from the 3D-data set and exemplarily shown for sheet nr. 9 (Fig. 7f). The data indicates that a high Signal-to-Noise-Ratio (SNR) could be obtained (SNR = 28 ± 4, av = 9). Also, the Contrast-to Noise-Ratio (CNR) related to the difference in the signal intensity of the liquid to the printer material is sufficient for the 8x printing (CNR = 22.1 ± 4.4).

The reconstructed data sets let the reader decipher the text pattern, thus demonstrating that MRM is in principle capable of resolving printed text. However, the data also shows:

1.) the importance of highest microscopic resolution with voxel size comparable to the printed letter thickness at sufficient SNR usually demanding for averaging with long measurement time;

2.) the relevance of the capability of 3D spatial processing software to reconstruct images along curved surfaces adjusted to the potentially bowed paper sheet surfaces. Results on this semi-automatic approach are shown in the next section.

### Improved, flattened visualization of the printed text on bowed paper sheets

As we have seen, page reconstruction based on rotated slices in the original 3D voxel data via even hyperplanes might not be sufficient, as the paper sheets might not be completely flat. Reconstruction along even hyperplanes therefore might give unsatisfactory results with limited text pattern recognition. The improvement available by semi-automatic positioning, reconstruction along bowed surfaces and automatic white balance adjustment is visualized in Fig. 8. Please compare overall visibility of the text patterns to Figs. 6d, 7c and 7f, all of which were obtained by manual positioning of even hyperplanes.

The same semi-automated process was also used to obtain optimally positioned hyperplanes to compare with manual positioning of slices. However, results were very similar to those visualized in Fig. 7 and are therefore not shown.

To obtain these results we significantly extended the algorithm described in^17^ to:

1.) semi-automatically track page surfaces, reducing significantly the efforts for manual positioning the hyperplanes and

2.) applied the collected data to fit parametric mathematical descriptions of the bowed pages.

We named the new algorithm TRIvial PAge TRAcker (or short TRIPATRA). It has a number of parameters which need to be carefully chosen to prevent tracking loss. Additionally, for each tracked page, one initial parameter needs to be given which defines an initial approximative center line positioned within the bowed page. As we focus on demonstrating principal feasibility here, a fully automated version is left for future work.

The application of TRIPATRA consists of seven steps, which are repeated for each consecutive slice. Page center line sets from the previously analyzed slice are taken as preliminary estimates for the new page center line set and then refined. A two-stage search process finds the page borders, starting from each pixel of each page center line. It removes obvious outliers, i.e., page borders that appear to indicate too thin or too thick pages. A robust RANSAC^18^ linear regression process operates on the whole set of page border samples. It enforces non-crossing of the estimated page surfaces and global page parallelism. RANSAC also estimates a new page center line set. This process is then repeated for the next slice.

For actual estimation of the page shape, we collected all of the 3D page center sample points generated by applying the TRIPATRA algorithm. We then used this data to fit several parametric models with linear weights. The high amount of about 300,000 to 400,000 samples per page made the fitting extraordinarily robust. As parametric models we fitted a small set of polynomial models on our data using a robust linear regression fitting algorithm (*lm* from R^19,20^), We assume the page is present in the *X/Y* plane and our page model (separately fitted for each page *p*) is therefore *Z* = *f*_*p*(*X, Y*)_ with *f*_*p*_ being a parametric function fitted to the noisy samples of the page center. We used the collected page center samples from all slices. 3D-hyperplanes are fitted using Eq. (1):

1 $${Z}_{{plane}}={a}_{0}+{a}_{1}* X+{a}_{2}* Y$$

The bowed surfaces can also be parametrized using a quadratic polynomial model (Eq. (2)):

2 $${Z}_{{quad}}={a}_{0}+{a}_{1}* X+{a}_{2}* Y+{a}_{3}* X* Y+{a}_{4}* {X}^{2}+{a}_{5}* {Y}^{2}$$

Finally, also a cubic model is used for fitting the bowed surfaces (Eq. (3)):

3 $${Z}_{{{{\rm{cubic}}}}} 	 = \; {a}_{0}+{a}_{1}* X+{a}_{2}* Y+{a}_{3}* X* Y+{a}_{4}* {X}^{2}+{a}_{5}* {Y}^{2} \\ 	 +{a}_{6}* {X}^{2}* Y+{a}_{7}* {Y}^{2}* \,X+{a}_{8}* {X}^{3}+{a}_{9}* {Y}^{3}$$

Although it would be expected that the page center samples perform better than either the blank back page or the printed front page, since they are averaged from front and back samples, this is not observed: all three variants effectively give indistinguishable results. This may possibly be explained by the very small variance in paper thickness for modern paper.

First, we fitted even hyperplanes (see Eq. (1)) to this data in order to estimate the best planar slice—not necessarily orthogonal to any axis—for each page. Page reconstruction was relatively bad. Only a small number of letters could be recognized (similar to Fig. 6 for the 2x printed letters (th ≈ 14 µm)). The results were very similar to those obtained by manual positioning – shown in Figs. 6 and 7 – and are therefore not shown.

Secondly, we fitted higher order polynomial models – quadratic (Eq. (2)) and cubic (Eq. (3)) models.

Both the quadratic and the cubic model showed better results, however, with the known propensity of higher-order polynomial models for overshooting, slightly more noticeably for the cubic model, especially in areas near the border. Although the difference between quadratic and cubic model was small, the cubic model showed slightly better results and was therefore chosen for visualization. Higher-order polynomial models yielded very similar results but the propensity for overshooting – especially outside the page – increased dramatically, so we stopped at cubic models.

The advantage of using models with so few parameters on such a large set of sample points is, that fitting is extremely robust and practically does not depend on tracking parameters, provided that page tracking does not fail completely. Furthermore, gaps in the page surfaces are automatically filled in. These models can fit all reasonably globally smooth page surfaces.

All considered models determine page shape. However, the optimal layer for visualization still needs to be determined. It should however lie near the printed front page. We, therefore, reprojected the original MRM dataset such that the computed page shape becomes exactly the X/Y plane, and determine the best position by visual inspection. For best results, we oversampled the pixel values by a factor of four, in effect using ¼ (quarter) interpolated subpixels along the Z axis. In most cases the position that yielded the best visual result was the one corresponding to the front side of the page where the actual text was written plus/minus half a pixel.

All images were automatically white-balanced to improve visibility, which stretches the pixel histogram for maximum contrast.

Concluding, Fig. 8 shows the page reconstruction results from the cubic model. The text can be read reasonably well. Air bubbles are present and – when large enough – occasionally prevent the recognition of single letters. Note, that the top row of images with twice toner thickness (th ≈ 14 µm) already shows distinct letters just at the border of visibility, which appears to represent a significant improvement over visualization along even hyperplanes (compare with Fig. 6d). This improvement in combination with a relatively small increase in SNR could potentially make this printing variant readable as well. Thus, the results of the semiautomatic surface flattening postprocessing of the MRM data set (Fig. 8, top row) outperform the results of the elaborate manual positioning (Fig. 6d) along 3D hyperplanes. Especially in the presence of MRT artefacts, the ability of parametric models to estimate page shape even in areas where no page surface samples exist makes them suitable for this kind of application.

## Discussion

### Summary

We found that MR-microscopy does allow for the measurement of printing paper thickness. The tiny elevations on the paper deposited by pigment-based inks during the printing process can be quantified in thickness using MR-active liquid add-ons as, for instance, silicone oil. However, the use of silicone oil might be not non-destructive and its removal might be harmful to sensitive objects. 3D-MR-microscopic pulse sequence protocols have to be used for the reading of short text patterns printed with minimum thickness of about 30 µm on several superposed bowed paper sheets. The 3D-protocols have to include small, close to isotropic voxel sizes which range down at minimum to the order of the print elevations, e.g., 30 µm. In order to be able to visualize the text patterns, the adjustment of display layers in the 3D-data set has to be performed by postprocessing and accurate alignment to the paper layers. Thus, the potential for MRM to “read books without opening” is demonstrated. However, the FOV and consequently the sample size was restricted within this experimental MRM demonstration of the physical technical principle by the diameter of the sensitive RF-detectors/-coils (about 19 mm). Larger FOVs demand mainly for multi-channel sensitive phased array RF-coils and longer measurement time.

MRM, as pointed out here, requires mainly the usage of high magnetic fields (B ≥ 7 T), strong magnetic field gradient systems (G ≥ 750 mT/m) for spatial encoding and sensitive RF-detectors. The essential resolution (e.g., isotropic VS ≤ (30 µm)^3^) can be achieved even on MR-scanners designed for human applications using an additional MRM gradient insert for MRM^8,10^ but might be also available on preclinical MR-scanners and experimental vertical MR-scanners designed for MR-microscopy, where an isotropic voxel size, e.g., of (3 µm)^3^ has already been obtained^9^.

### Comparison to other potential text reading microimaging techniques

Standard X-ray based Computer tomography (CT) relies with regard to contrasting letters on the difference in the absorption of X-rays between the information carrying print or ink material and the surrounding paper or medium, e.g., air. Using µ-CT scanners already larger scale books (e.g., 17 × 17 × 3 cm^3^) containing letters with metal can be visualized at isotropic (103 µm)^3^) voxel size. Best results are obtained on inks with metal content or, more generalized, with elements with high atomic number Z due to the more efficient photoelectric effect. Even higher spatial resolution might be obtained for imaging with commercially available µ-CT scanners but the visualization of inks containing no metals remained difficult^21,22^. For instance, the carbon-based ink on Herculaneum paper rolls could not be differentiated from the carbonized papyrus in CT scans^23^.

The problem of “unfolding”, i.e., 2-Dimensional (2D) display of letters on bowed or folded paper sheets, e.g., in un-opened letters has been addressed up to now mainly on µ-CT (XMT) data^24,25^. The “letter locking” algorithm uses an XMT scanner with 668 dpi, i.e., 38 µm nominal pixel size.

Phase Contrast X-ray Imaging (PCI), based on differences in X-ray propagation with phase alterations in the investigated object, has been proposed for obtaining contrast also for molecules with atoms of low atomic number. Different measurement approaches have been presented for soft tissue X-ray contrast in non-invasive biomedical imaging, e.g., Propagation-Based phase contrast (PBI) and analyzer-based imaging, crystal and grating interferometry, scattering and dark field imaging^26^. However, all of these PCI techniques demand for advanced prototype equipment adjusted individually for being successfully applied to the visualization of printed letters on paper with non-metallic ink. This is due to the high challenges on spatial resolution, contrast, penetration of hidden objects and unrolling phase contrast being present on bowed surfaces. Adapted prototype high resolution PCI methods have to fulfill different severe requirements on X-ray beam like monochromaticity, size of X-ray source for spatial coherence, optical stability, X-ray intensity, and experimental conditions like object-to detector-distance and object size^26^.

For more detailed information on methodological aspects and some recent results of phase contrast X-ray-imaging on ancient Herculaneum papyri with carbon based ink and usage of trained AI routines for enciphering ancient text the reader is recommended to read the attached supplementary information (section “Comparison to other potential text reading microimaging techniques”).

Also, perspectives for X-ray absorption-based contrast and µ-CT with added contrast agents are discussed there.

In principle also particle beams with low matter interaction can be used for the deciphering of text or ancient symbols without destruction. Using neutron tomography Wilster-Hansen et al.^27^ recently demonstrated that runic symbols on a medieval amulet could be visualized. The amulet consists of folded lead sheets, which usually cannot be penetrated by X-ray and likely will present challenges to MRM. However, the high-resolution neutron tomography with nominal resolution of about 30-50 µm, was limited with regard to the FOV by the detector size (27 mm)^2^. This high-resolution neutron tomography demands for unique equipment (ICON) in connection with the spallation neutron source SINQ at PSI Villigen/CH.

THz-CT (0.3 THz) has been demonstrated to show potential for the imaging of covered letters^28^. Three letters (“THz”) at about 30 mm size and 1 mm thickness had been written with ultramarine blue color on canvas, rolled up in a spiral form. The letters could be deciphered after removing metal artifacts and unfolding processing. However, THz-CT is restricted in spatial resolution by the physical resolution connected to the wavelength (λ ≅ 1 mm for f = 0.3 THz) and scanning step width, significantly less than for MRM or µ-CT. The penetration depth of THz electromagnetic waves decreases with higher frequency, which might limit applications especially for metallic or water containing samples. With advantage to µ-CT the photon energies used are significantly smaller than for X-ray CT which reduces the risk for material degrading. The photon energies of MRM are still about 3 orders of magnitude less than those of THz-technology.

A recent review on different approaches for imaging of hidden text including X-ray- fluorescence and - tomography, Infrared tomography, THz and Photoacoustic imaging, ion beam analysis and neutron radiation for digital recovery of heritage offers a more detailed overview on recent advances in non-MRI based methods^29^.

### Actual limitations of the presented MRM approach with regard to methodology and applications

#### Potential invasiveness using silicone oil as MR-active medium

The adding of an MR active liquid or gas is necessary in the indicated MRM based approach in order to obtain an MR-signal and contrast against letters offering no-signal.

The removal of silicone oil as MR-active liquid after MR visualization may be destructive to antique books, sensitive paper or parchment. There are some alternatives for silicone oil, which are shortly discussed in the attached supplementary file section “Potential invasiveness using silicone oil as MR-active medium” for interested readers. We did not perform additional experiments on verification. The potential of using other MR-active media or modified UTE sequences should be explored also by experimental proof-of-concept in future studies.

#### Limitations in MRM technology with regard to “state-of-the-art” magnetic resonance scanners and applications

The reading of closed large books using MRM is not possible yet, due to mainly two opposing requirements:

- large Field of View (FOV) with open magnet bores of min. 40 ×40 cm^2^ incl. gradient hardware for spatial encoding. The large bore is necessary for covering the book dimension;
- high sensitivity necessary for obtaining the high spatial resolution.

However, already available MR-Hardware technology might be combined in a prototype MR-microscopy unit for large FOV optimized for deciphering printed letters in closed books taking advantage of (a) modern gradient construction hardware, (b) improvement in the signal to noise ratio (SNR) using sensitive small-sized rf-coil arrays and (c) artificial intelligence (AI) supported detection of letter-induced slight signal variations in MRM data, being hardly recognized by the human vision system as already proposed for µ-CT^23^.

For a more detailed discussion on already available hardware and potential further improvements the interested reader is referred to the attached supplementary material, section “Limitations in MRM technology with regard to “state of the-art” magnetic resonance scanners and applications”.

### Perspectives for applications in other scientific disciplines

The low-invasive type of measurement using Magnetic Resonance Imaging principles might not only be used for reading of valuable text patterns but also allows in principle for the visualization of images and sculptures, covered and not directly accessible from outside. The method relies on elevated forms on underlying even or slightly bowed layers, e.g., non-colored indentations in stone or wood, for instance ancient clay tablets. Especially in archeology or even criminology 3D MRI and MRM might offer deciphering and slice viewing in arbitrarily selected orientations inside an object. However, an MR signal active liquid, which might have impact on the investigated object is to be added for high resolution visualization in most cases.

**Fig. 9:**
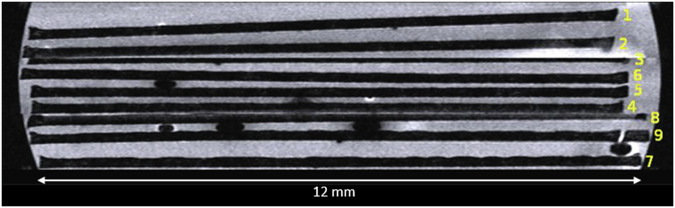
Lateral MR-view of the paper pages stacked over each other indicating their positions referring to the views of Fig. 8. Positioning of pages within the test sample (xz slice, *y* = 446 as in Fig. 7) but rotated by 180° around the y-axis so that sheet 1 is on top – and contrast-enhanced via white-balancing – see text). Pages appear black, and space between pages (MR-active contrast medium) appears white. Recording was done separately for sheets 1-3, 4-6 and 7-9, and then stitched together with MRT recording software. Artefacts between the three recordings are due to imperfect stitching, which also caused printed page surface of sheets 3 and 8 to be partially missing, so no usable page surface could be obtained there.

## Supplementary information

Supplementary Information

## Data Availability

The datasets generated during and/or analysed during the current study for Figs. 1 to 7 are available from A.B., and data sets for Figs. 8, 9 and Supplementary Fig. S1 are available from A.S. on reasonable request.

## Code availability

The implementation of TRIPATRA (Section “Improved, flattened visualization of the printed text on bowed paper sheets”) and the projection code to extract bowed paper sheets according to the parameters estimated from its output is available upon reasonable request from the co-author A.S. (programming language: Java). The provided implementation will include the necessary parameters including the initial page lines to fully reproduce all images from the given 3D MRM dataset.
