# Supplementary material for: Visualization of text on bowed sheets via High-resolution 3D-Magnetic Resonance Micro-imaging for potential reading of closed books: the proof-of-concept
Source: Commun Eng. 2026 Feb 25;5:71. doi: 10.1038/s44172-026-00614-7 (PMC13079842; doi:10.1038/s44172-026-00614-7)
Supplement: Supplementary file 1 — Supplementary Information [file 44172_2026_614_MOESM1_ESM.pdf]

Visualization of text on bowed sheets via High-resolution 3D-Magnetic Resonance Micro-imaging for potential reading of closed books: the proof-of-concept
Supplementary Information

Andreas Georg Berg[1,2*]

[1] Medical University of Vienna, Center for Medical Physics and Biomedical Engineering, Waehringer Guertel 18-20, A-1090 Vienna, Austria
andreas.berg@meduniwien.ac.at
[2] Medical University of Vienna, High Field Magnetic Resonance Center, Lazarettg. 14, A-1090 Vienna, Austria

Alexander Karl Seewald[3]

[3] Seewald Solutions GmbH, Lärchenstraße 1, A-4616 Weißkirchen a.d. Traun, Austria
alex@seewald.at

I Supplementary Introduction and Content Overview

This supplementary material mainly adds a more detailed description referring to the chapter II Material and Methods, the section on the subject of MR methodology: *2.3.2 MRM-microscopy apparatus, MR-pulse sequences and -protocols* and the chapter IV Discussion, sections: *4.2 Comparison to other potential text reading microimaging techniques and 4.3 Actual limitations of the presented MRM approach with regard to methodology and applications*

Subsequently, a summary of the different subsections is offered to the reader for deciding which subsection may be read in more details:

4.2 Comparison to other potential text reading microimaging techniques

Here, a more detailed non-complete discussion on methodological aspects of absorption based µCT and phase contrast X-ray-imaging is given. Some interesting recent imaging applications on ancient Herculaneum papyri with carbon-based ink and usage of trained AI routines for enciphering ancient text are reported.

Also, perspectives for X-ray and µCT with added liquid or gas agents for improved contrast are discussed here.

4.3 Actual limitations of the presented MRM approach with regard to methodology and applications

4.3.1 Potential invasiveness using silicone oil as MR-active medium. Here methods for removal of the silicon oil and alternatives as MR-active media are shortly discussed.

4.3.2 Limitations in MRM technology with regard to "state of the-art" magnetic resonance scanners. This section contains a discussion on already available hardware for a) human applications with diameters available for even larger books and b) sensitive MR-microscopy systems with small available diameters but offering in principle sufficient high spatial resolution. In addition, potential further improvements for achieving a full low-invasive deciphering of closed books are indicated.

Index Terms: Magnetic Resonance Imaging, Microscopy, µCT, phase contrast X-ray imaging, text, letter, archeology, low-invasive

II Supplementary Material and Methods

2.3 MRM-microscopy apparatus, MR-pulse sequences and -protocols

2.3.1 Challenges and concepts

The physical-technical demands refer mainly to the small voxel volumes necessary for identification of the very thin letter structure, which represent challenges for the sensitivity of the RF-detectors and the spatial encoding in the direction of the letter thickness[1].These challenges have been addressed using:

a) prototype hardware add-ons on an Ultra-High Field (UHF) human MR-scanner ($B = 7$ T); These are mainly relying on a distinctly stronger magnetic gradient field than available on a clinical MR-scanner (factor 10) and a sensitive RF-detector with small size adapted to the sample diameter.

b) a time efficient 3D-FT pulse sequence and MR-protocols adapted to the MR-active liquid along with averaging of signal for Signal-to-Noise-Ratio (SNR) improvement.

2.3.2 Hardware

The prototype hardware add-on to an UHF MR-scanner for human investigations mainly consists of a strong gradient system $G = 750$ mT/m, which is about a factor 10 stronger than usually available for human MR-scanning but restricted to an available inner diameter for the RF-coil and the sample of i.d. = 9 cm[2,3]. The strong gradient system is necessary for separating the spectral RF-signal for neighboring voxels beyond the inherent spectral width of the MR-signal[1].

For addressing the problem of the small number of molecules in the tiny microscopic voxel size the RF-sensitivity is distinctly increased using small sample-size adapted (inner available diameter: e.g., i.d. = 19 mm), cylindric type, quadrature driven resonator for RF transmission and reception ($f = 297.2$ MHz, Rapid Biomedical, Würzburg, Germany). The maximum Field of View (FOV) for this RF-coil is restricted to 19 mm. An adapter box with preamplifier is interfacing the RF-detector

with the standard clinical MR plug system box of the UHF human MR-scanner (*Siemens 7T Magnetom*). The whole microscopy-insert including gradient system, RF-coil and interface is located on the patient table and positioned in the Isocenter of the MR-scanner[2,3] before starting the measurement. The insert is connected to an MR-microscopy PC-console, separated from the human scanner console for safe operation. Measurement protocol parameters are set up using Siemens standard console software (*Syngo VE 12U*).

2.3.3 MR pulse sequences and MRM protocols

Initial MRI experiments using gradient echo imaging (Fl2D1) revealed tiny air bubbles connected to the surface of the paper sheets. Besides from missing MR signal from the corresponding regions, these air bubbles feature a different magnetic susceptibility than water or silicone oil, which results in spatial encoding artefacts in MRI. The gradient echo imaging techniques are more sensitive to these distortions. Consequently, spin-echo techniques with reduced susceptibility related artifacts have been used. Silicone oil features high T_2 values ($T_2 \cong 300$ ms). For time efficient spatial encoding a Turbo-Spin Echo sequence (TSE) with up to ten phase encoding steps (TSE3D1_10, Turbo-factor 10) within one RF-excitation has therefore been used. The small microscopic voxel volumes still demand for additional signal enhancement, which we achieved by an increased number of repetitive, signal-intensity added measurements (averages) at the expense of measurement time. Several different protocols have been used to optimize the results for the different sample sets. The most important MRM -protocols used for the results shown in Fig. 2 - Fig. 9 are listed in Supplementary Table S1.

**Prot.-name**	**2D-Localizer$_{sag}$**	**3D-iso60**	**3D-iso20**	**3D-iso20$_{large}$**
Figure	2	3	4	6-9
Pulse sequ.	tse2D1_5	tse3D1_10	tse3D1_8	tse3D1_10
Turbo-factor	5	10	8	10
Voxel size [µm^3]	20x20x200	58x59x60	20x20x20	18l x 18l x 20
FOV [mm^3]	6 x 6 x 11.4	5 x 7.6 x 1.1	5 x 5 x 3.8	14.2 x 16 x 3.8
MTX	256x256x29	86x128x18	256x256x192	804lx896lx192
TE [ms]	12	12	17	15
TR [ms]	2000	2000	2000	5000
Averag.	1	1	12	9
Measurement time TM [min]	2	6	1851	2223
Band-width bw [Hz]	296	200	201	199
Source file	2019_11_4_4	2019_11_4_5	2019_11_4_11	2019_12c_10

Supplementary Table S1 MR-protocol parameters for 2D- and 3D-MR-microscopy on single printed letters, single and multi-stacked sheets of printed paper (l:interpolated)

2.3.4 Postprocessing of 3D data sets for slice selective imaging

The 3D-time domain data sets with frequency and phase encoded spatial information is automatically decoded by 3D-Fourier transform on the Siemens *Syngo* platform of the MR-scanner with slice orientation according to measurement protocol set-up. However, these slices do often not fit sufficiently accurate to the layer of the printed paper sheet. For accurate positioning for reading the text patterns the 3D-FT data set arranged in separate files for the different slices was read into a public domain image processing tool (ImageJ V1.53t)[4]. The multi-slice data is visualized in arbitrary selected orientations using the Image J plugin package "volume viewer"[5]. The position of the viewing slice was adjusted manually in an iterative procedure to the layer of the text pattern using the orthogonal viewing modality of the volume viewer.

2.3.5 Calculation of Signal-to Noise (SNR and Contrast to-Noise Ratios CNR)

SNR of an image is calculated here as signal intensity I_{liquid} in a ROI related to a volume filled with the MR-active liquid close to a central letter (e.g., M in Fig. 7):

The noise number N for an image is defined and calculated as standard deviation ($s_{Icorner}$) of the signal intensity (I_{corner}) outside of the sample (air) in the corner of the images:

$$SNR = I_{liquid} / \langle s_{Icorner} \rangle \qquad (1).$$

The relevant information on contrast is given by the difference of the signal intensities in the MR-active liquid (I_{liquid}) and the low signal from the print material (I_{print}). Consequently, we defined similar to the *Weber contrast* definition:

$$CNR = I_{liquid} - I_{print} / \langle s_{Icorner} \rangle \qquad (2).$$

IV SUPPLEMENTARY DISCUSSION

4.2 Comparison to other potential text reading microimaging techniques

X-ray based Computer tomography (CT) relies - with regard to contrasting letters - on the difference in the absorption of X-rays between the information carrying print or ink material and the surrounding paper or medium, e.g., air. Using µ-CT scanners already larger scale books (e.g., 17 x 17 x 3 cm^3 containing letters with metal can be visualized at isotropic (103 µm)^3 voxel size. Best results are obtained on inks with metal content or, more generalized, with elements with high atomic number Z due to the more efficient photoelectric effect. Even higher spatial resolution might be obtained for imaging with commercially available µ-CT scanners but the visualization of inks containing no metals remained difficult[6,7]. For instance, the carbon-based ink on Herculaneum paper rolls could not be differentiated from the carbonized papyrus in CT scans[8].

The problem of "unfolding", i.e., 2-Dimensional (2D) display of letters on bowed or folded paper sheets, e.g., in un-opened letters has been addressed up to now mainly on µ-CT (XMT) data[9,10]. The "letter locking" algorithm uses an XMT scanner with 668 dpi, i.e., 38 µm nominal pixel size.

Phase Contrast X-ray Imaging (PCI), based on differences in X-ray propagation with phase alterations in the investigated object, has been proposed for obtaining contrast also for molecules with atoms of low atomic number. Different

measurement approaches have been presented for soft tissue X-ray contrast in non-invasive biomedical imaging, e.g., Propagation-Based phase contrast (PBI) and analyzer-based imaging, crystal and grating interferometry, scattering and dark field imaging[11].

Recently X-ray dark field imaging[12] has been applied at low photon energy ($U = 30$ kV) for visualizing non-metallic hand-writing. The method is based on X-ray phase contrast changed by the sample instead of using the absorption contrast by implementing 2 grids between the sample and the detecting CMOS array (effective pixel size in the object plane: 32.3 µm). The visualization of a hand-written word ("Gescheit") on a single paper sheet produced according to the medieval recipe for thorn ink had been demonstrated. The ink does not contain any metallic components. However, the authors do also indicate that an envelope or a stack of superposed sheets of paper "pose an additional challenge, since every further layer of paper adds more background on the X-ray images".

PBI has been applied recently to carbonized rolls of ancient Herculaneum papyri with carbon-based ink, offering no elements with high X-ray absorption. Single letters, a coronis symbol used to mark the end of a textual section and small portions of text could be deciphered relying on differences in the carbon fiber structure in the papyrus rolls and the ink[13].

Deciphering 5% of text on a Herculaneum carbonized scroll has been achieved within a price donated competition using tiny "crackle" contrast in µ-CT images that seemed to appear like Greek letters and machine-learning algorithms (trained AI routines)[8].

However, all of these PCI techniques demand for advanced prototype equipment adjusted individually for being successfully applied to the visualization of printed letters on paper with non-metallic ink. This is due to the high challenges on spatial resolution, contrast, penetration of hidden objects and unrolling phase contrast being present on bowed surfaces. Adapted prototype high resolution PCI methods have to fulfil different severe requirements on X-ray beam like monochromaticity, size of x-ray source for spatial coherence, optical stability, X-ray intensity, and experimental conditions like object-to detector-distance and object size[11].

The MRM based deciphering of printed text (MRM-reading) as demonstrated here is based on the elevation of the printed text above the layer of paper. In principle µ-CT should be able to detect elevations on paper too, if there is sufficient thickness of an air layer (offering absorption contrast in Hounsfield-units between air and printed letter on top of the paper). However, this separate air layer might not be present in overlying gravity pressed paper sheets as also potentially for MRM based methods using liquids. Previous experiments using µ-CT on the differentiation had not been successful. We had recorded a mini-book (printed in 1950 using lead letters) with X-ray µ-CT with a voxel size of 6 µm, but - while pages and even single paper fibers were clearly visible - we had been unable to detect any text (see Supplementary Figure S1).

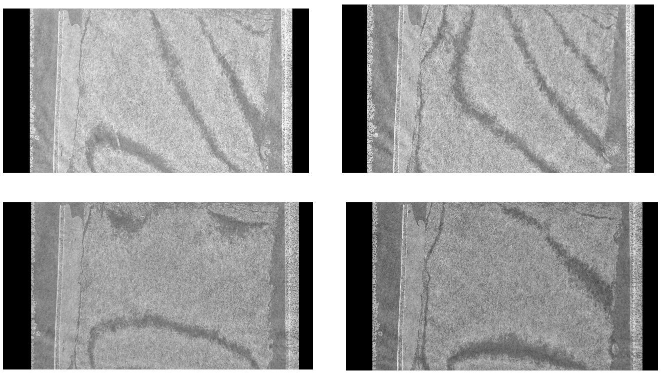

Supplementary Figure S1: µ-CT image of text on paper sheet. Microbook "Poem for Newly Wedded Couples" (1950 edition), 10x8.5mm, printed with lead letters. The first four text pages were manually extracted from the X-ray µCT recording of the whole book. Resolution / voxel size was 6µm. No text is discernable.

We indicate here that µ-CT might also be used with liquids or gas containing elements with high atomic nr. for generating increased absorption contrast.

The MRM-reading contrast against paper using an MR-active medium (here silicone oil) could presumably also be established on a) penetration differences of the MR-visible agent between paper and ink and b) mobility differences of the liquid within paper vs. ink (e.g., via T_2 or diffusivity of the liquid in the two media, due, e.g., to carbon fiber orientation similar to the origin of phase contrast in µ-CT as indicated in[13]. Thus, MRM-reading might not only be relying on the elevation of letters beyond the paper level. However, we are not able to experimentally test all of the different modifications of MRM-reading within this initial demonstration of the capabilities of MRM using silicone oil.

X-ray imaging in principle uses photon energies capable of breaking chemical bindings and consequently the X-ray dose applied to the material represents potential for damages to delicate samples[14]. The dose has to be controlled and limited as pointed out also by the authors for avoiding those damages, a potential problem to the visualization of whole books demanding for a higher number of projections. X-Ray imaging usually cannot be used for thicker metal plates due to the high absorption, a potential challenge also for MRM as it relies on the emission of radio-waves which could be Faraday-caved by metal enclosing.

In principle also particle beams with low matter interaction can be used for the deciphering of text or ancient symbols without destruction. Using neutron tomography Wilster-Hansen et al.[15] recently demonstrated that runic symbols on a medieval amulet could be visualized. The amulet consists of folded lead sheets, which usually cannot be penetrated by X-ray and likely will present challenges to MRM. However, the high-resolution neutron tomography with nominal resolution of about 30-50 µm, was limited with regard to the FOV by the detector size (27 mm)[2]. This high-resolution neutron - tomography demands for unique equipment (ICON) in connection with the spallation neutron source SINQ at PSI Villigen/Ch.

THz-CT (0.3 THz) has been demonstrated to show potential for the imaging of covered letters[16.] 3 letters ("THz") at about

30 mm size and 1 mm thickness had been written with ultramarine blue color on canvas, rolled up in a spiral form. The letters could be deciphered after removing metal artifacts and unfolding processing. However, THz-CT is restricted in spatial resolution by the physical resolution connected to the wavelength ($\lambda \cong 1$ mm for $f = 0.3$ THz) and scanning step width, distinctly less than for MRM or μ-CT. The penetration depth of THz electromagnetic waves decreases with higher frequency, which might limit applications especially for metallic or water containing samples. With advantage to μ-CT the photon energies used are distinctly smaller than for X-ray CT which reduces the risk for material degrading. The photon energies of MRM are still about 3 orders of magnitude less than those of THz-technology.

A recent review on different approaches for imaging of hidden text including X-ray- fluorescence and - tomography, Infrared tomography, THz and Photoacoustic imaging, ion beam analysis and neutron radiation for digital recovery of heritage offers a more detailed overview on recent advances in non-MRI based methods[17].

4.3 Actual limitations of the presented MRM approach with regard to methodology and applications

4.3.1 Potential invasiveness using silicone oil as MR-active medium

The adding of an MR active liquid or gas is necessary in the indicated MRM based approach in order to obtain an MR-signal and contrast against letters offering no-signal.

The removal of silicone oil as MR-active liquid after MR visualization may be destructive to antique books sensitive paper or parchment. There are some alternatives for silicone oil, which are discussed here. However, we did not perform additional experiments on verification. The potential of using other MR-active media or modified UTE sequences should be explored also by experimental proof of concept in future studies.

The first approach with water exhibited no obvious impact on printed ink or paper, however resulted in air bubble distortions and MRI artifacts. Silicone oil is known to be chemically rather inert, featuring low viscosity for penetrating even into thin slits between paper sheets and openings of μm size. The appearance of air-bubbles and connected imaging artefacts is strongly reduced with reference to water. No additional contrast agent with potential chemical impact to delicate samples is added. The silicone oil is available with different viscosities and boiling off temperatures and might be removed after imaging using higher temperatures and/or vacuum or solid/liquid separation using centrifuges. Also, other MR-active liquids or even 1H-atoms containing gas at high pressure might be used. The most critical feature of this MR-active add-on is represented by the demands presented by small voxel size and consequently sensitivity: high density of MR-active atoms (e.g., 1H or 19F) and T_2 higher than about 2 ms for Ultrashort Time Encoding (UTE) methods $T_2 \geq 200$ μs[18,19]. As an alternative perspective, hyperpolarized noble gas or PARA-hydrogen[20,21] transferred chemically to liquids with distinctly higher magnetic polarization might be used as MR-active agents. However, these alternative MR active agents are difficult to be prepared, demand for additional hardware equipment and present other limitations like restrictions in measurement time. These are beyond the scope of this initial presentation demonstrating the principal detectability of printed letters using MRM along with silicone oil as MR-active medium. The applicability of other MR-active media for different sensitive objects are to be proved by future object specific experiments.

4.3.2 Limitations in MRM technology with regard to “state of the-art” magnetic resonance scanners and applications

The reading of closed large books using MRM is not possible yet, due to mainly two opposing requirements:

a) large Field of View (FOV) with open magnet bores of min. 40 x 40 cm^2 incl. gradient hardware for spatial encoding. The large bore is necessary for covering the book dimension;

b) high sensitivity necessary for obtaining the high spatial resolution.

We wish to compare here two different types of "state of the art" MR-scanners, which are optimized for these two limiting demands, with the prototype MRM-system as used for the MRM-reading approach here: a) Ultra-High-Field (UHF) MR-scanners optimized for investigating humans with large cross areas up to about 40x40 cm^2 along with high sensitivity[22,23] and b) MR-microscopy systems optimized for highest resolution.

a) UHF ($B_0 \geq 7$T) scanners for human imaging are commercially available and used also in clinical routine with sensitive multi-channel head coil detectors. They feature high magnetic fields in order to increase sensitivity characterized by the Signal-to-Noise-Ratio (SNR $\propto B_0^{7/4}$)[24]. They are equipped with strong magnetic field gradients G for spatial encoding in humans, typically G $\simeq$ 80 mT/m[25]. Using such systems along with a multi-array radiofrequency detector system composed of 31 single small RF-coils a human brain can be visualized at nominal spatial resolution with voxel-size VS = (100 μm)^3 within long measurement time: (TM = 100 h![26]. However, this resolution is not sufficient for resolving printed text with thickness th $\leq$ 30 μm as indicated here. Ultra-High-field prototype MR-scanners have been put into operation for in-vivo head imaging demonstrating a voxel size of 190 x 190 x 1000 μm^3 at $B = 11{,}7$ T in acquisition time below 5 min[27]. MR-scanners are projected for even higher magnetic fields ($B = 14$ T)[28] offering sufficient FOV for imaging on humans.

b) MR-microscopy systems optimized for highest resolution

Commercial MR-microscopy systems are available at even higher magnetic fields ($B > 7$T) ranging up to about 17.6 T[29] and as state-of-the-art prototype system at $B = 28$ T for preclinical imaging, e.g., on zebrafish as an animal model for neuronal pathology[30]. They offer stronger hardware for even higher magnetic field gradients up to about $G = 1000$ mT/m[29] and in conceptual designs even $G = 2450$ mT/m[31] for separating neighbouring voxels by frequency or phase encoding on the cost of smaller inner diameter (di $\leq$ 6 cm) available for objects to be investigated. They do also offer sensitive small sized surface rf-detector coils (size $\simeq$ cm). In preclinical imaging on rats and mice voxel sizes of about (30 μm)^2 x 200 μm can be

obtained in-vivo[32] and 23 x 23 x 100 μm^3 on Zebrafish brain[30]. Using cryo-rf-coils[33] (sensitive radiofrequency-detectors) cooled down to temperatures of a few °K for reducing electronic noise an even higher spatial resolution with isotropic voxels of (33 μm)^3 could be obtained in TM = 6 h[32].
Even higher spatial resolutions with voxels smaller than (10 μm)^3 are in principle possible using prototype ultra-sensitive micro-rf-coils with diameters of 70-120 μm, e.g., 3.7x3.4x3.3μm^3 (TM ≃ 30h)[34,35] operated at room temperature.
These approaches with small-sized sensitive detector exhibit the potential for sufficient high resolution to detect very thin printed letters but are limited with regard to the Field-of -View (FOV) and sample size to the detector size (d ≃100 μm).
Consequently, the imaging of letters in closed books of larger size than about 2 cm as shown within this report (FOV = 1.6 cm, di = 1,9 cm) using a prototype microscopy insert to a human MR-scanner appears to be not available yet by existing "state of the art" MR-scanners.
For comparison, within the presented MRM approach, we implemented a microscopy insert on a human UHF MR-scanner using a strong gradient system with available inner diameter of 9 cm. The diameter for objects to be scanned is additionally reduced by the sensitive rf-coil (with available inner diameter of di ≃ 19 mm. We proved that the detection of 30 μm thick text patterns is possible within the silicone oil added medium approach. Even single 15 μm thin 2x printed letters could potentially be recognized using semiautomatic additional postprocessing of the 3D MRM data along bowed paper sheet surfaces using an MR-active liquid.

4.4 Potential hardware improvements for low invasive reading of books

Already available MR-Hardware technology might be combined in a prototype MR-microscopy unit optimized for deciphering printed letters in closed books taking advantage of a) modern gradient construction hardware, b) improvement in SNR and c) artificial intelligence (AI) supported detection of letter induced signal variations in MRM data.
We would like to consider an example for estimation of necessary equipment: a book of thickness th_B = 5 cm lateral size: W x L = 20 x 20 cm^2.
The gradient strength necessary for separating the signal of neighbouring pixels by their emitted radio-frequency in the most challenging z-direction (the direction of the assumed elevation direction of printed letters) can be calculated from the subsequent equations[36,1].

$$G = \Delta f/(\gamma\, \Delta z) \qquad (6)$$

Δz: thickness of letters e.g,, th = 8 μm;
γ: gyromagnetic ratio (γ_{1H} = 42.58 MHz/T;
Δf: minimum frequency separation between neighbouring pixels in spatial encoding by emitted frequency determined by the spectral width (sw) of the MR-signal of the liquid (e.g., sw = 60 Hz, measured for the silicone oil).

Inserting the required spatial resolution (Δz = 8 μm), and spectral width (Δf = sw = 60 Hz) in equ. (6) yields a minimum gradient strength: $G_{sw} \geq 176$ mT/m for separating neighbouring voxels by radiofrequency.
Such gradient systems are already commercially available for head MR-scanner systems[23]. Even whole-body human MR-scanners offer a gradient strength of G = 200 mT/m with an inner open diameter sufficient for human investigations with RF-coils for patients[37]. Prototype gradient systems for head scanning with available inner diameter of 44 cm are reported working at G = 500 mT/m[38].

The necessary sensitivity of RF-detectors along with the required large FOV might be achieved using small cm-sized rf-coil array systems, with single size and sensitivity similar to the prototype microscopy insert used within this report. This multi-channel receiver detector with 64 separate receiver channels using sensitivity encoding and parallel imaging[39] might reduce scanning time offering still high sensitivity. In addition, cryocooling of this array system including preamplifiers might increase sensitivity by a factor 2.3-5.3[33], which can be used for enhancing resolution in z-direction, detecting voxels with smaller z-extension down to 8 μm (20 μm as used in the protocol here, divided by factor 2.5).
AI routines, similar as indicated for μ-CT on papyrus scrolls[24], for detecting the tiny "crackle" type modifications in image contrast may be used for deciphering letters, which are even hard to be recognized by human visual inspection.
The penetration of books by MR-active liquids with very closely connected pages still has to be investigated by experiment.
Therefore, dedicated MRM devices for reading books might be capable of detecting printed text on larger FOVs in future. On a longer time-scale other methods for sensitivity enhancement, e.g., based on hyperpolarization[40] might represent the option for future solutions with distinctly shorter measurement time of the contradictory challenges combining large FOV and high sensitivity for necessary spatial resolution. However, these approaches represent other limitations in application and demand also for problem adapted imaging equipment.

SUPPLEMENTARY References

1. Callaghan, P.T. *Principles of Nuclear Magnetic Resonance Microscopy*. Oxford Clarendom Press, (2003).

2. Berg, A., Potthast, A., Starewicz, P. MR-MICROSCOPY on a human 7T-SCANNER. In: Proc. ISMRM/ESMRMB 2010, 1048 Stockholm, Sweden (2010).

3. Berg, A.G. and Börner, M. A phantom for the quantitative determination and improvement of the spatial resolution in slice-selective 2D-FT magnetic resonance micro-imaging and -microscopy based on

Deep X-ray Lithography (DXRL). *Front. Phys.* 11:1144112 (2023). https://doi.org/10.3389/fphy.2023.1144112.

4. Wayne Rasband and contributors National institutes of Health, USA Java 1.8.0_245 (64 bit); https://imagej.nih.gov/ij/; acc. 20.09.2023).

5. Plug-in “volume viewer”; Kai Uwe Barthel (barthel at htw-berlin.de) Internationale Medieninformatik, HTW Berlin, Germany (2023). https://imagej.net/ij/plugins/volume-viewer; acc. 19.12.2025.

6. Stromer, D., Christlein, V., Martindale, C. et al. Browsing through sealed historical manuscripts by using 3-D computed tomography with low-brilliance X-ray sources. *Sci Rep* 8, 15335 (2018). https://doi.org/10.1038/s41598-018-33685-4.

7. Jaggard, V., (20 January 2015). Ancient Scrolls Blackened by Vesuvius Are Readable at Last. Smithsonian. https://www.smithsonianmag.com/history/ancient-scrolls-blackened-vesuvius-are-readable-last-herculaneum-papyri-180953950/; acc. 28.08.2025.

8. Marchant, Jo (5 February 2024). First passages of rolled-up Herculaneum scroll revealed. Nature. 626 (7999): 461–462 (2024). https://doi.org/10.1038/d41586-024-00346-8.

9. Dambrogio, J., Ghassaei, A., Smith, D.S. et al. Unlocking history through automated virtual unfolding of sealed documents imaged by X-ray microtomography. *Nat Commun* 12, 1184 (2021). https://doi.org/10.1038/s41467-021-21326-w.

10. Stromer, D., Christlein, V., Huang, X. et al. Virtual cleaning and unwrapping of non-invasively digitized soiled bamboo scrolls. *Sci Rep* 9, 2311 (2019). https://doi.org/10.1038/s41598-019-39447-0.

11. Bravin, A., Coan, P., & Suortti, P. X-ray phase-contrast imaging: from pre-clinical applications towards clinics. *Physics in medicine and biology*, 58(1), R1–R35 (2013). https://doi.org/10.1088/0031-9155/58/1/R1.

12. Akstaller, B., Schreiner, S., Dietrich, L., Rauch, C., Schuster, M., Ludwig, V., Hofmann-Randall, C., Michel, T., Anton, G., & Funk, S. X-ray Dark-Field Imaging for Improved Contrast in Historical Handwritten Literature. *Journal of Imaging*, *8*(9), 226 (2022). https://doi.org/10.3390/jimaging8090226.

13. Bukreeva, I., Mittone, A., Bravin, A. *et al.* Virtual unrolling and deciphering of Herculaneum papyri by X-ray phase-contrast tomography. *Sci Rep* **6**, 27227 (2016). https://doi.org/10.1038/srep27227.

14. Mantler M , Klikovits J. Analysis of art objects and other delicate samples: is XRF really nondestructive? *Powder Diffr* **19** 16–19 (2004).

15. Wilster-Hansen, B., Mannes, D.C., Holmqvist, K.L., Ødeby, K., Kutzke, H. Virtual unwrapping of the BISPEGATA amulet, a multiple folded medieval lead amulet, by using neutron tomography. *Archaeometry*. 64(4), 969-978 (2022). https://doi.org/10.1111/arcm.12734.

16. Tianyi Wang, Kejia Wang, Kaigang Zou, Sishi Shen, Yongqiang Yang, Mengting Zhang, Zhengang Yang, Jinsong Liu. Virtual unrolling technology based on terahertz computed tomography. *Optics and Lasers in Engineering*, **151**, 106924 (2022). https://doi.org/10.1016/j.optlaseng.2021.106924.

17. Perino, M., Pronti, L., Moffa, C., Rosellini, M., Felici, A.C. New Frontiers in the Digital Restoration of Hidden Texts in Manuscripts: A Review of the Technical Approaches. *Heritage* 7, 683–696 (2024). https://doi.org/10.3390/heritage7020034.

18. Robson, M.D., Gatehouse, P.D., Bydder, M., Bydder, G.M. Magnetic resonance: an introduction to ultrashort TE (UTE) imaging. *J Comput Assist Tomogr*. 27(6), 825-46 (2003). https://doi,org/10.1097/00004728-200311000-00001.

19. Berg, A. G., Hatamikia, S., Valladares, A., Ptacek, W., Kronreif, G., Lorenz, A. High-field MRI of Polymer/Silicone material for body compartment phantoms and interventional devices: Visibility with Standard GRE MR protocols, UTE-imaging and quantitative T1/T2 determination. Proc. Joint Conference of the ÖGMP, DGMP & SGSMP Dreiländertagung Medizinische Physik 2021,eP21; 159-160 (2021).

20. Bowers, C. R., Weitekamp, D. P. Parahydrogen and synthesis allow dramatically enhanced nuclear alignment. *J. Am. Chem. Soc.* **109**, 5541–5542 (1987).

21. Eisenschmid, B.C., Kirss, R. U., Deutsch, P. P., Hommeltoft, S. I. Eisenberg, R., Bargon, J., Lawler, R. G., Balch, A. L. , *J. Am. Chem. Soc.* **1987,** *109,* 8089–8091.

22. Siemen Healthineers, Medical Imaging, Magnetic Resonance Imaging, 7T MRI Scanner, Magnetom Terra; section Technical Details. https://www.siemens-healthineers.com/en-us/magnetic-resonance-imaging/7t-mri-scanner/magnetom-terra; acc. 03.09.2025.

23. GE Healthcare, MRI, neuroimaging, SIGNA MAGNUS, 09. July 24 in IMAGING Product News "GE HealthCare unveils groundbreaking head-only

MRI scanner". https://interhospi.com/ge-healthcare-unveils-groundbreaking-head-only-mri-scanner/; acc. 04.09.2025.

24. Mansfield, P., Morris, P.G., NMR IMAGING IN BIOMEDICINE in *Advances in MAGNETIC RESONANCE* Suppl. 2, ed. J.S. Waugh, Academic Press, Inc Orlando (1982).

25. Siemen Healthineers, Medical Imaging, Magnetic Resonance Imaging, 7T MRI Scanner, Magnetom Terra; section Technical Details; https://www.siemens-healthineers.com/en-us/magnetic-resonance-imaging/7t-mri-scanner/magnetom-terra; acc. 03.09.2025.

26. Edlow, B.L., Mareyam, A., Horn, A. et al. 7 Tesla MRI of the ex vivo human brain at 100 micron resolution. *Sci Data* 6, 244 (2019). https://doi.org/10.1038/s41597-019-0254-8.

27. Boulant, N., Mauconduit, F., Gras, V., Amadon, A., Le Ster, C., Luong, M., Massire, A., Pallier, C., Sabatier, L., Bottlaender, M., Vignaud, A. & Le Bihan, D. 2024. In vivo imaging of the human brain with the Iseult 11.7-T MRI scanner. *Nature Methods*, 21, 2013-2016.

28. Eisenstein, M. 2024. Pushing the limits of MRI brain imaging. *Nature Methods*, 21, 1975-1979.

29. Bruker Bruker.com. Products and solutions, preclinical imaging, Billerica: Bruker Corporation. (2022). https://www.bruker.com/en/products-and-solutions/preclinical-imaging/mri/biospec-ultra-high-field-mri.html; acc. sept 01, 2025.

30. Singer, R., Oganezowa, I., Hu, W. B., Ding, Y., Papaiooannou, A., De Groot, H. J. M., Spaink, H. P. & Alia, A. Unveiling the Exquisite Microstructural Details in Zebrafish Brain Non-Invasively Using Magnetic Resonance Imaging at 28.2 T. *Molecules*, 29(19), 4367 (2024).

31. He, H., Wang, Y., Wang, Z., Wei, S., Wang, H., Liu, F., Yang, W. & Wang, Q. 2025. A hybrid gradient coil design method generating ultra-high gradient magnetic field for micro-MRI utilization. *Review of Scientific Instruments*, 96, 104702 (2025).

32. Kelm, N. High resolution morphology imaging of mouse and rat brain in Bispec Brochure; Bruker.com. Products and solutions, preclinical imaging, Billerica: Bruker Corporation. https://www.bruker.com/en/products-and-solutions/preclinical-imaging/mri/biospec-ultra-high-field-mri.html; acc. sept 01, 2025.

33. Bruker Preclinical MRI CryoProbes. https://www.bruker.com/en/products-and-solutions/preclinical-imaging/mri/MRI-CryoProbes.html; acc. 04.09.2025.

34. Ciobanu L, Seeber DA, Pennington CH. 3D MR microscopy with resolution 3.7 µm by 3.3 µm by 3.3 µm. *J Magn Reson* 158:178–82 (2002). doi:10.1016/s1090-7807(02)00071-x.

35. Lee, S.C., Kim, K, Kim, J., Yi, J.H., Lee, S., Cheong, C. MR microscopy of micron scale structures. *Magn Reson Imaging* **27**, 828–33 (2009). doi:10.1016/j.mri.2009.01.002.

36. Glover P, Mansfield P. Limits to magnetic resonance microscopy. *Rep. Prog Phys* **65**:1489–511 (2002).

37. Siemens Healthineers, Medical Imaging, Magnetic Resonance Imaging, Upgrades, Magnetom Cima.X Fit; section Unprecedented gradient strength; https://www.siemens-healthineers.com/magnetic-resonance-imaging/options-and-upgrades/upgrades/magnetom-cima-x-fit#unprecedented-gradients-strength; acc. 04.09.2025.

38. Huang, Susie Y et. al. Connectome 2.0: Developing the next-generation ultra-high gradient strength human MRI scanner for bridging studies of the micro-, meso- and macro-connectome. *NeuroImage* **243** 118530 (2021). doi:10.1016/j.neuroimage.2021.118530.

39. Pruessmann, K.P., Weiger, M,, Scheidegger, M.B,, Boesiger, P. SENSE: sensitivity encoding for fast MRI. *Magn Reson Med*. **42**(5):952-62 (1999). PMID: 10542355.

40. Kuhn, Lars T.; et al., eds. Hyperpolarization methods in NMR spectroscopy. Berlin: Springer (2013). ISBN 978-3-642-39728-8.
